# Intramedullary schwannoma of the upper cervical spinal cord: a case study of identification in pathologic autopsy

**DOI:** 10.1080/20961790.2016.1265236

**Published:** 2017-01-25

**Authors:** Xianxian Li, Guangtao Xu, Ruibing Su, Junyao Lv, Xiaoping Lai, Xiaojun Yu

**Affiliations:** a Medicolegal Department, Shantou University Medical College, Shantou, China; b Department of Pathology, Jiaxing University Medical College, Jiaxing, China; c Department of Forensic Medicine, Guangdong Medical University, Dongguan, China

**Keywords:** Forensic science, forensic pathology, asphyxia, spinal cord, intramedullary schwannoma

## Abstract

Intramedullary schwannoma of the upper cervical spinal cord is rarely reported in forensic medicine. We herein report a case involving a patient who died of compression from an intramedullary schwannoma in the upper cervical spinal cord. A 30-year-old man initially presented with a five-day history of pain in the left chest that progressed to weakening in the left arm. Although the patient was treated with analgesic poultices, he developed inspiratory dyspnoea and died while working the next day without having undergone any medical imaging examination or surgical treatment. Anatomical and histopathological examinations revealed an intramedullary schwannoma in the left cervical spinal cord (C_3_–C_5_) underneath the spinal nerve root. The cause of death might have been asphyxia secondary to the tumour, which interfered with the nerve function in the respiratory muscles. This finding suggests that an autopsy is essential for pathologists and medicolegists to comprehensively undertake their due obligation to obtain “the first evidence”, especially when there is a lack of directly related evidence. As part of the central nervous system, the spinal cord could be systematically included in a routine pathological autopsy in some cases.

## Introduction

Schwannomas account for 30% of primary intraspinal tumours [1]. They mainly originate from nerve sheaths in the spinal canal and are usually located in the extramedullary intradural and/or extradural regions. With regard to intramedullary schwannomas, evidence has shown that approximately 0.3% and 1.1% of intramedullary schwannomas are intraspinal tumours and intraspinal schwannomas, respectively [2]. Intramedullary spinal schwannomas are rare. Since the earliest report in 1931, only 67 cases have been reported. Most of them were detected in the clinical setting, and after confirmation of the diagnosis, they were resected successfully [3–6]. In general, these tumours progress slowly and require surgical treatment. We herein report a case of a rapidly progressing schwannoma that resulted in death, possibly due to asphyxia based on the autopsy and histopathological findings.

## Case report

### Case history

A 30-year-old man was admitted to a private clinic with a five-day history of pain in the left chest and progressive weakness in the left arm. The pain was typically experienced as a paroxysmal dull ache that worsened after activity. The patient had no history of similar symptoms. He consulted with a general practitioner, and analgesic poultices were applied as symptomatic treatment to alleviate the pains. However, the patient developed dizziness during work the next day. He subsequently developed inspiratory dyspnoea without any other clinical symptoms, and he was ultimately confirmed dead by emergency physicians.

### Autopsy findings

A systemic pathological autopsy was performed for histopathological examination and judicial poisoning analysis. Nebulous and reddish lividity were anomalously distributed on the back. The face, anterior neck and upper chest exhibited diffuse ecchymosis. Petechial haemorrhage was found on the bulbar and palpebral conjunctiva, showing signs of asphyxia. The lips, fingernails and toenails showed cyanosis. No bleeding or fractures were observed throughout the body including in the craniocerebrum; neck, chest and abdominal subcutaneous tissues; hyoid bone; thyroid cartilage; cricoid cartilage and trachea.

**Figure 1.**
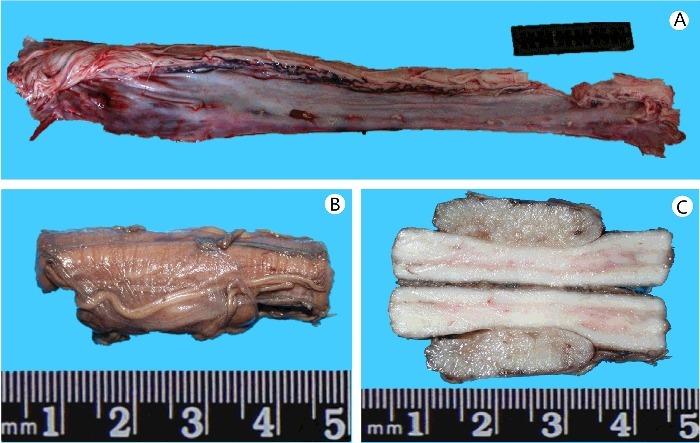
Macroscopic view of the intramedullary schwannoma. (A) The neoplasm in the cervical spinal cord (C_3_–C_5_). (B) The long, oval neoplasm (2.5 cm × 1.0 cm × 1.0 cm). (C) Local concave compression of C_3_–C_5_.

**Figure 2.**
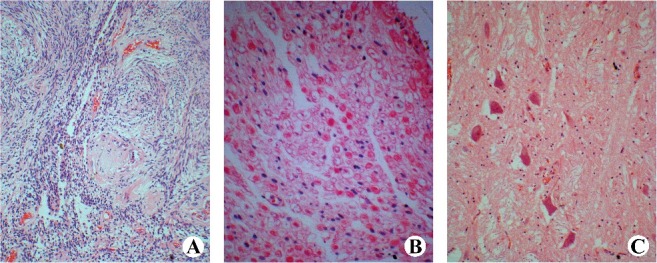
Microscopic examination of the intramedullary schwannoma. (A) Verocay body (HE ×400). (B) Hollow-like degeneration of the spinal nerve tracts (HE ×200). (C) Hydropic degeneration of anterior spinal cord motor neurons (HE ×400).

A neoplasm measuring 2.5 cm × 1.0 cm × 1.0 cm was located in the left cervical spinal cord (C_3_–C_5_) underneath the spinal nerve root, with a clear boundary and integrated surface of the neoplastic capsule (Figure 1). The compressed spinal cord was visibly sunken. Microscopic examination revealed widespread and recurrent haemorrhage, haemosiderin particle aggregation and scattered infiltration of lymphocytes within the subarachnoid space of the C_3_–C_5_ spinal cord. The tumour cells exhibited a long spindle shape, were uniform in size and had a fence-shaped, whirlpool, wavy arrangement. Sporadic Verocay bodies (Antoni type A), haemangiectasis with paralytic congestion, myxoid matrix deposition and vascular wall hyaline degeneration (Antoni type B) were present within the tumour (Figure 2(A)). The axons of the ventral root of the spinal nerve were unevenly thick and homogeneously solid, and they exhibited hollow degeneration (Figure 2(B)). Netlike and loose spinal cord parenchymal nerve fibres were irregularly and hyperchromatically enlarged, distorted and fractured, and they extended to the outer space. Hydropic degeneration and lipofuscin pigment deposition were observed in ventricolumnar motor neurons (Figure 2(C)). The pathological diagnosis was a cervical intramedullary spinal cord schwannoma. The neurons exhibited slight oedema, and no necrocytosis was found in the compressed spinal cord and brain stem. The other organs and tissues exhibited diffuse hydropic degeneration. The peripheral blood, gastric contents, urine and hepatic tissue tested negative by conventional toxicology analysis.

## Discussion

Schwannomas are best known as benign intraspinal tumours located in the subdural and epidural spaces [7–9]. The cervical cord (58%) is the most commonly affected site, followed by the thoracic cord (32%) and lumbar cord (10%) [10]. The tumour is derived from Schwann cells, which produce the nerve sheath. Schwannomas differ depending on their nerve origin and location within the head and neck [11]. There are two types of histopathological changes. Antoni type A is characterized by the presence of tumour cells in a close-knit fascicular arrangement, and Antoni type B is characterized by intercellular structures arranged in a loose mesh-like structure [12–17]. Intramedullary schwannomas have no specific imaging features, and magnetic resonance imaging is the most useful method for diagnosis of spinal intramedullary tumours. Therefore, a correct preoperative definitive diagnosis is pivotal for the treatment and prognosis. The pathogenesis of intramedullary schwannomas remains controversial because the central nervous system contains no Schwann cells. Thus, case reports can add useful information for further understanding.

In the present case, no clinical examination was performed and no clear diagnosis was made, making it difficult for the pathologists and medicolegists to obtain full information regarding death. The evidence we acquired was mainly from the autopsy and histopathological examination. The asphyxial symptoms were conspicuous in the ecchymotic face, anterior neck and upper chest. The bulbar and palpebral conjunctiva exhibited petechial haemorrhage. The lips, fingernails and toenails were cyanotic. Meanwhile, the craniocerebrum; neck, chest and abdominal subcutaneous tissues; hyoid bone; thyroid cartilage; cricoid cartilage and trachea showed no bleeding or fractures. Toxicological analysis was negative. Based on these findings, mechanical asphyxia and poisoning could be excluded. The pathological autopsy in this case indicated that Antoni type A was the main component of the C_3_–C_5_ intramedullary schwannoma, which was accompanied by acute repressive oedema and necrosis in the local spinal cord and nerve root. The phrenic nerves, which are composed of C_3_–C_5_ spinal nerves and dominate the diaphragmatic muscle movement, can promote abdominal respiration [18,19], while neural injury can contribute to inspiratory dyspnoea and ultimately asphyxia. To the best of our knowledge, schwannomas are benign tumours, and intramedullary spinal schwannomas have a slow growth pattern. The average interval between the appearance of first symptoms and diagnosis is 36.6 months (range, 1.5 months to 20.0 years) [3]. Due to the anatomical specificity and complexity, the spinal canal is wider at the cervical aspect than at the other parts, especially in the upper cervical region, which has no cervical enlargement. This feature provides a larger space for tumour growth, and the tumours are always quite large before symptoms appear, resulting in the subsequent development of severe nerve dysfunction, motor disturbances and respiratory dysfunction. The tumour in the present case was located in the left cervical spinal cord (C_3_–C_5_) and measured 2.5 cm × 1.0 cm × 1.0 cm, and the patient died within a short period of time. We consider that the large range of spinal motion during his work may have aggravated the spinal compression. The diaphragmatic muscle cannot contract normally because of the neural injury secondary to compression by an upper cervical intramedullary spinal cord schwannoma, eventually leading to asphyxia.

Intramedullary schwannomas can give rise to luminal stenosis and obstruct the cerebrospinal fluid circulation, which decreases absorption in the lumbosacral portion. In addition, tumour metabolites and constriction of the spinal cord can induce increased permeability of the blood vessels to the extent that the protein components increase, which blocks arachnoid granulation and further progresses into reactive basicranial arachnoiditis, resulting in a barrier to cerebrospinal fluid circulation [16,17,20,21].

Another report in 2013 described a patient who died of brain stem compression induced by intratumoural haemorrhaging within an intracranial schwannoma, showing that brain stem compression can result in respiratory disturbances [22]. The definite diagnosis and location make it goal-directed to find the cause, while the herein-reported cases, for which this information was unavailable, prove the importance of spinal cord examination in forensic practice. No unified pathological autopsy protocol has been established. Autopsy generally includes cranial, thoracic, and abdominal and pelvic cavity examinations; spinal cord examination is not yet considered an essential element of autopsy except in some cases involving obvious neurological symptoms such as headache, nausea and vomiting, altered mental status and papilloedema [23]. The spinal cord can be collected from either the anterior or posterior route. Compared with the posterior route, the anterior route is more easily affected by artificial factors because of the anatomical characteristics and the state of the corpse. The anterior route has obvious artificial factors that cannot be avoided. Therefore, we adopted the posterior route in the present case; more time is required, but the results are more informative and accurate [24]. In addition, despite the nonspecific neurological symptoms in this case, our conventional approach including post-mortem spinal cord inspection was beneficial for identification of the cause of death. This case clarifies that without exception, a full and complete examination that includes the spinal cord and ribs should be the rule in every case [25]. Only in this way can we avoid missing primary and important related information. We suggest to pathologists and medicolegists who undertake their due obligation of collecting “the first evidence” that the spinal cord, as part of the central nervous system, should be systemically included in routine pathological autopsies.
